# Augmenter of liver regeneration ameliorates renal fibrosis in rats with obstructive nephropathy

**DOI:** 10.1042/BSR20140038

**Published:** 2014-09-05

**Authors:** Guo-tao Chen, Ling Zhang, Xiao-hui Liao, Ru-yu Yan, Ying Li, Hang Sun, Hui Guo, Qi Liu

**Affiliations:** *Department of Nephrology, The Second Affiliated Hospital, Chongqing Medical University, Chongqing 400010, China; †Institute for Viral Hepatitis, Key Laboratory of Molecular Biology for Infectious Diseases, The Second Affiliated Hospital, Chongqing Medical University, Chongqing 400010, China

**Keywords:** augmenter of liver regeneration, renal fibrosis, Smads protein, transforming growth factor-β1, tubular epithelial–mesenchymal transition, ALR, augmenter of liver regeneration, CKD, chronic kidney disease, ECM, extracellular matrix, EMT, epithelial–mesenchymal transition, GAPDH, glyceraldehyde 3-phosphate dehydrogenase, HGF, hepatocyte growth factor, IgG, immunoglobulin G, rhALR, recombinant human ALR, RT, reverse transcription, TGF-β, transforming growth factor β, UUO, unilateral ureteral obstruction

## Abstract

Renal fibrosis is a hallmark in CKD (chronic kidney disease) and is strongly correlated to the deterioration of renal function that is characterized by tubulointerstitial fibrosis, tubular atrophy, glomerulosclerosis and disruption of the normal architecture of the kidney. ALR (augmenter of liver regeneration) is a growth factor with biological functions similar to those of HGF (hepatocyte growth factor). In this study, our results indicate that endogenous ALR is involved in the pathological progression of renal fibrosis in UUO (unilateral ureteral obstruction) rat model. Moreover, we find that administration of rhALR (recombinant human ALR) significantly alleviates renal interstitial fibrosis and reduces renal-fibrosis-related proteins in UUO rats. Further investigation reveals that rhALR suppresses the up-regulated expression of TGF-β1 (transforming growth factor β1) induced by UUO operation in the obstructed kidney, and inhibits Smad2 and Smad3 phosphorylation activated by the UUO-induced injury in the animal model. Therefore we suggest that ALR is involved in the progression of renal fibrosis and administration of rhALR protects the kidney against renal fibrosis by inhibition of TGF-β/Smad activity.

## INTRODUCTION

CKD (chronic kidney disease) has received increasing attention as a leading public health problem with rapid growth in its prevalence. The overall incidence of CKD is 10.8% in China [1]. It is well established that renal tubular injury, renal interstitial inflammatory cell infiltration and renal interstitial fibrosis occur during the progression of CKD [2]. Renal fibrosis is a pathological hallmark of CKD, which is characterized by tubulointerstitial fibrosis, tubular atrophy, glomerulosclerosis and disruption of the normal architecture of the kidney. It strongly correlates with the deterioration of the renal function, and ultimately leads to ESRD (end-stage renal disease) [3].

In the literature reported previously, renal fibrosis is the consequence of the activation and accumulation of fibroblasts, and the deposition of abundant ECM (extracellular matrix) that occurs in virtually every type of the CKD [4]. Fibroblasts are mesenchymal cells residing or embedded in the ECM of connective tissues and organs. They can secrete proteins, particularly the molecular collagen that forms the extracellular fibrillar matrix of connective tissue. Renal myofibroblasts appear *de novo* in renal fibrosis, and they most probably represent a stressed and dedifferentiated phenotype of the fibroblasts [5]. Numerous studies have shown that the progressive renal fibrosis is mediated by the growth factors, cytokines, metabolic toxins and stress molecules via complex mechanisms and multiple pathways. Among these mediators, TGF-β1 (transforming growth factor β1) has been recognized as a key promoter in the pathogenesis of renal fibrosis [6–8]. Meanwhile, some other mediators are found to exert marked anti-fibrotic effects in the pathogenesis of renal fibrosis, such as HGF (hepatocyte growth factor) [9], EPO (erythropoietin) [10] and BMP-7 (bone morphogenetic protein-7) [11]. Recent studies have shown that these mediators display therapeutic potential against renal fibrosis *in vivo* [12–14]. Hence, exploring and investigating additional anti-fibrotic mediators may have clinical significance and contribute to the treatment of renal tubulointerstitial fibrosis in CKD.

ALR (augmenter of liver regeneration) is a growth factor with biological functions analogous to those of HGF [15,16]. It is found to be expressed in liver [17], testis [18] and kidney [19]. Current research on ALR mainly focuses on its biological function in the liver, and we rarely find reports on ALR's function in other viscera. Our previous studies have confirmed that ALR expression increases in acute kidney injury in rat, and intraperitoneal injection of exogenous recombinant ALR shows obvious renal protective function in ischaemia reperfusion rat model [20]. However, it is not clear if ALR is involved in the pathophysiological process of chronic damage of renal tubulointerstitium.

In this study, we investigated whether the administration of rhALR (recombinant human ALR) affected the pathophysiological process of renal fibrosis in UUO (unilateral ureteral obstruction) animal model. UUO is a well-established experimental method for renal disease, which results in tubulointerstitial fibrosis in animal models [21,22]. In UUO rat model, we observed the change of endogenous ALR expression, indicating its involvement in the pathological progression of renal fibrosis in the obstructed kidney. Moreover, rhALR significantly ameliorated the tubulointerstitial fibrosis and reduced the expression of proteins associated with renal fibrosis in UUO rats.

## MATERIALS AND METHODS

### Establishment of UUO model and rhALR treatment regimen

Sprague–Dawley adult male rats (250–300 g body weight) were housed under 12 h light/dark cycle, and were allowed free access to food and water. The rat model of obstructive nephropathy was induced by UUO: a midline incision was made in the abdominal wall, the left ureter was dissected out and ligated with 4.0 silk suture at two points along its length. The abdominal wound was closed with the same silk suture and the animals were returned to the cages [23]. Rats were randomly divided into five groups: (1) sham control group; (2) sham+rhALR 200 μg/kg/day group; (3) UUO+PBS; (4) UUO+rhALR 100 μg/kg/day; (5) UUO+rhALR 200 μg/kg/day (courtesy of Institute for Viral Hepatitis, Chongqing Medical University, Chongqing). rhALR was dissolved in PBS and administered to rats via daily intraperitoneal injection after UUO surgery according to the indicated dose. All rats were killed on days 3, 7 and 14 after UUO or sham operation, and the kidney tissue was collected for various analyses. Animal experiments were performed in accordance to the regulations set by the Institutional Committee for the care and use of laboratory animals. The protocol had been approved by the Animal Care and Research Committee of Chongqing University of Medicine.

### Histology and immunofluorescence

For histological analysis, kidney tissues fixed with 4% (v/v) buffered paraformaldehyde were embedded in paraffin, and 4-μm-thick sections were prepared. The sections were then stained with HE (haematoxylin and eosin) and Masson's trichrome.

Immunofluorescence analysis was carried out using anti-ALR polyclonal antibody, anti-α-SMA monoclonal antibody (Santa Cruz Biotechnology) and anti-vimentin monoclonal antibody (AbCam). Cryostat sections (4-μm thick) were fixed in cold acetone, and then permeabilized with 0.4% (v/v) Triton X-100 for 14 min, blocked in 5% (v/v) goat serum for 60 min and then incubated with different primary antibody overnight at 4°C in refrigerator, followed by the incubation with FITC-labelled anti-rabbit IgG (immunoglobulin G) (Zhongshan Goldenbridge Biotechnology) for 90 min. The slides were visualized by confocal laser-scanning microscopy (TCS-SP2, Leica). In each experimental setting, immunofluorescent images were captured with identical light exposure time.

### Semi-quantitative assessment of renal fibrosis

Kidney sections were prepared at 4-μm thickness and stained with Masson's trichrome for light microscopic examination. Five different randomly selected regions on the stained sections (three paraffin sections prepared from each kidney) were analysed by BX43 Clinical Microscope (Olympus America Inc). Fibrosis was graded by two independent pathologists. According to the Banff quantitative criteria for interstitial fibrosis [24], the extent of fibrosis in the renal cortical area up to 5, 6–25, 26–50% and more than 50% were graded as score 0, 1, 2 and 3, respectively.

### Real-time PCR

Total RNA was extracted using Trizol (Invitrogen). The quality RNA was examined by integrity of 28S and 18S rRNA on agarose gel and by A260/A280 ratio. RT (reverse transcription) of the RNA was performed using the Reverse-Transcription System (Takara). Using the cDNA mixture and primers, real-time RT–PCR was performed on an ABI Prism 7300 (Applied Biosystems) with SYBR Green polymerase chain reaction master mix (Takara Korea Biomedical). The primer sequences used were as follows: vimentin, forward 5′-AGGCAAAGCAGGAGTCAAACG-3′ and reverse 5′-TTCTCTTCCATTTCACGCATCTG-3′; α-SMA, forward 5′-TGCTGGACTCTGGAGATG-3′ and reverse 5′-GTGATCACCTGCCCATC-3′; fibronectin, forward 5′-CA-GGCTCAGCAAATCGTGCA-3′ and reverse 5′-CCCCAC-GACCTAGGAAGTC-3′; collagen I: forward 5′-GCCTCCC-AGAACATCACCTA-3′ and reverse 5′-ATGTCTGTCTTGCC-CCAAGT-3′; and β-actin, forward 5′-TCAGGTCATCACTA-TCGGCAAT-3′ and reverse 5′-AAAGAAAGGGTGTAAA-ACGCA-3′.

These primers were obtained from Takara. The PCR reaction was evaluated by the melting curve analysis. PCR was performed in duplicate and three independent experiments were performed. β-Actin was used as internal control.

### Western blot analysis

Renal tissue (0.1–0.2 g) from the rats was lysed with a homogenizer at 3000 rpm in a solution containing 250 mM sucrose, 1 mM EDTA, 0.1 mM PMSF and 20 mM potassium phosphate buffer at pH 7.6. Equal amounts of protein (20 μg/lane) were fractionated by SDS–PAGE electrophoresis. After being transferred onto a PVDF membrane (0.25μm) at constant voltage, the blots were probed with rabbit polyclonal anti-ALR (Santa Cruz Biotechnology; 1:200 dilution), rabbit monoclonal anti-GAPDH (glyceraldehyde-3-phosphate dehydrogenase) (Santa Cruz Bio-technology; 1:500 dilution), anti-collagen I (Santa Cruz Biotechnology; 1:1000 dilution), anti-fibronectin (Santa Cruz Biotechnology;1:800 dilution), anti-Smad2 (AbCam; 1:1500 dilution), anti-p-Smad2 (Bioworld Technology;1:800 dilution), anti-Smad3 (AbCam; 1:2000), anti-E-cadherin (AbCam; 1:2000 dilution), anti-TGF-β1 (AbCam; 1:2500 dilution) and rabbit polyclonal anti-p-Smad3 antibodies (Cell Signaling Technology; 1:1000 dilution). The membranes were incubated with primary antibodies at 4°C overnight. After being washed in TBST (Tris-buffered saline containing Tween 20) for three times, the membrane was incubated with HRP (horseradish peroxidase)-tagged goat anti-rabbit IgG (1:500) for 1 h (Santa Cruz Biotechnology). Proteins were visualized by chemiluminescence with the ECL (enhanced chemiluminescent) substrate (Genechem). GAPDH was used as internal control for each membrane.

### Statistical analysis

All data were expressed as the mean±S.D. Statistical analysis was carried out using the SPSS 17.0 software. Comparison between groups was made using one-way ANOVA followed by the Student–Newman–Kuels test. *P*<0.05 was considered to be statistically significant.

## RESULTS

### The expression of endogenous ALR in rat UUO kidney

**Figure 1:**
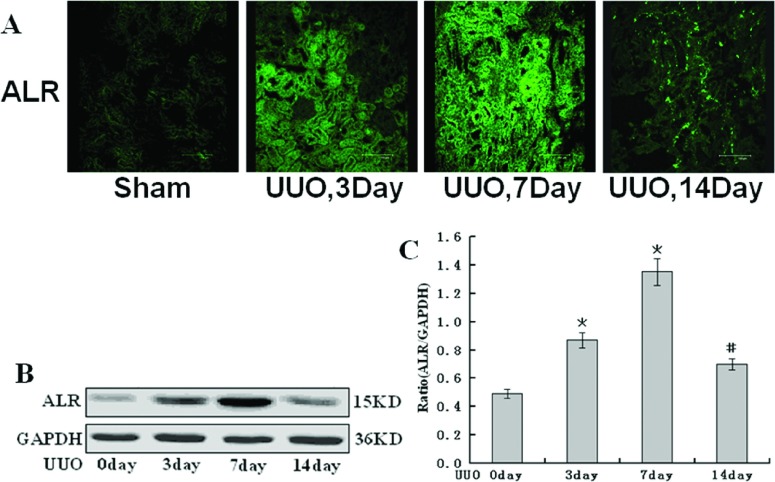
The expression of endogenous ALR in the obstructed kidneys underwent UUO in rats (**A**) Laser confocal immunofluorescence staining analysis shows that the expression of endogenous ALR present a small amount (barely detectable) in the kidney in the sham-operated group, gradual increases in the kidney 3, 7 days after UUO, but render decreases in the kidney 14 days after UUO. (**B**) Western blot analysis show that the expression of endogenous ALR present gradual increases in the kidney 3, 7 days after UUO, but decreases in the kidney 14 days after UUO. (**C**) Graphical presentation of relative expression of endogenous ALR. Values are presented as the density of ALR versus GAPDH (%). Mean±S.D. (*n*=6) were obtained from the densitometric analysis of all individual experiments.*P<0.05 versus Sham group. #*P*<0.05 versus UUO 7 days.

We examined the expression of endogenous ALR in the obstructed kidneys of the UUO rats. We observed the low expression of endogenous ALR in the tubulointerstitium and no ALR expression in the glomerulus in the sham-operated kidney tissue by immunofluorescence staining. There was significant increase in the expression of endogenous ALR in the UUO kidneys on days 3 and 7 after the surgery. However, the expression of ALR declined in UUO kidneys 14 days after the surgery (Figure 1A). Furthermore, the similar results were obtained using Western blot analysis (Figures 1B and 1C).

### Effects of rhALR on renal histology in rat UUO model

**Figure 2:**
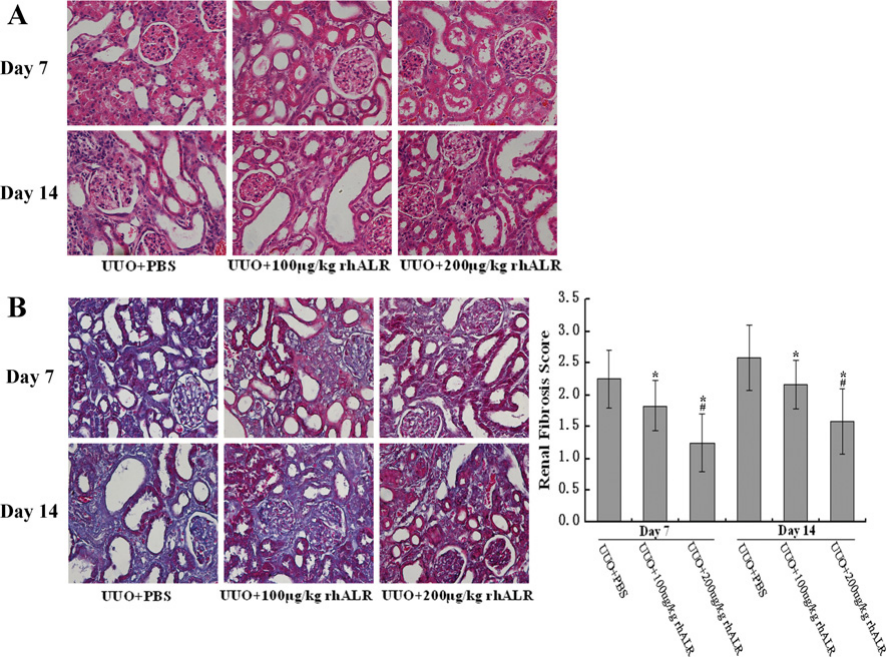
Treatment with rhALR suppresses interstitial fibrosis HE-stained (**A**) and Masson's trichrome-stained (**B**) paraffin-embedded rats kidney sections. (**C**) Semiquantitative-score of tubulointerstitial fibrosis in the cortex of the kidneys. Data are the mean±S.D. of four randomfields of three paraffin sections prepared from each kidney (*n*=6 in each group). *P<0.05 versus UUO+PBS; #*P*<0.05 versus UUO+100μg/kg/day rhALR. Original magnification, ×400.

We further assessed the effects of rhALR on injury-induced renal fibrosis *in vivo*. Histological examination showed obvious interstitial fibrosis represented by the excessive ECM accumulation such as strong collagen deposition in the obstructed kidneys. However, the obstructed kidneys treated with 100 or 200 μg/kg/day of rhALR displayed lower degree of interstitial fibrosis (Figures 2A and 2B). Semi-quantitative analysis on days 7 and 14 after UUO surgery revealed significantly lower fibrosis score in the obstructed kidneys treated with 200 μg/kg/day rhALR than in those treated with PBS (Figure 2C). These results demonstrated rhALR's ability to prevent UUO-induced renal fibrosis in a dose-dependent manner *in vivo*.

### Effects of rhALR on the proteins involved in renal fibrosis in rat UUO model

**Figure 3:**
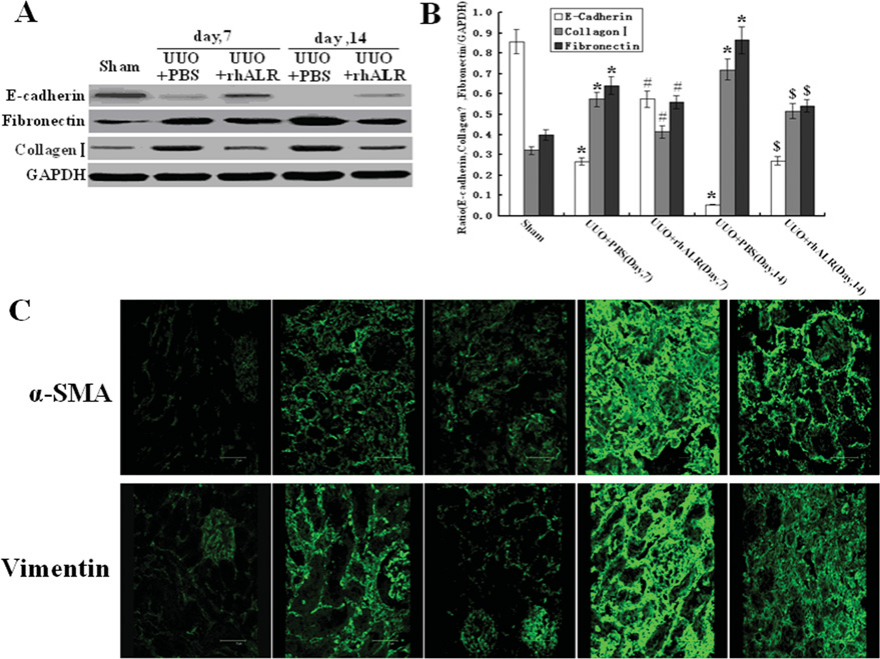
Effects of rhALR on the protein and/or gene expression of E-cadherin, vimentin, α-SMA, fibronectin and collagen I in the obstructed kidneys in UUO rats (**A**) Western blot analysis shows 200 μg/kg/day rhALR can decrease the protein expression of fibronectin and collagen I and increase the expression of E-cadherin in the obstructed kidneys on days 7 and 14 after UUO. (**B**) Graphical presentation of relative expression of E-cadherin, fibronectin and collagen I. Values are presented as the density of target proteins versus GAPDH (%). Mean±S.D. (*n*=6) were obtained from the densitometric analysis of all individual experiments. (**C**) Laser confocal immunofluorescence staining analysis shows that 200 μg/kg/day rhALR can decrease the protein expression of vimentin and α-SMA in the obstructed kidneys on days 7 and 14 after UUO surgery. *P<0.05 versus Sham group. #*P*<0.05 versus UUO+PBS (day 7). $*P*<0.05 versus UUO+PBS (day 14).

**Figure 4:**
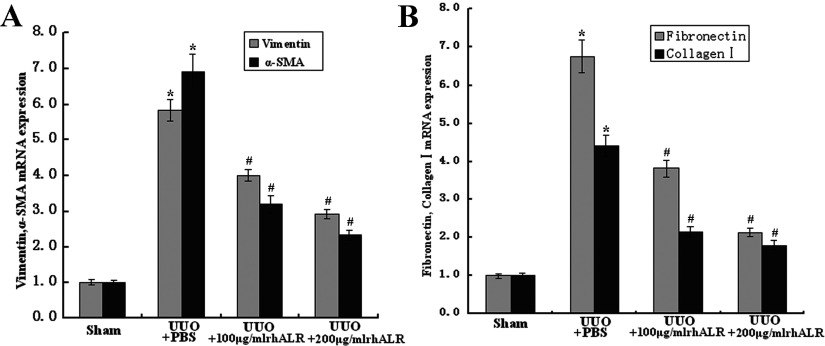
Effects of rhALR on the gene expression of vimentin, α-SMA, fibronectin and collagen I in the sham-operated and obstructed kidneys 14 days after UUO surgery, measured by real-time quantitative PCR β-Actin is used for normalization. *n*=6, mean±S.D. *P<0.05 versus Sham group. #*P*<0.05 versus UUO+PBS.

We also investigated the expression of proteins related to renal fibrosis, such as E-cadherin, vimentin, α-SMA, fibronectin and collagen I in PBS- or rhALR-treated rat UUO kidneys. Western blot analysis demonstrated that rhALR significantly up-regulated the protein expression of E-cadherin, and down-regulated the protein expression of fibronectin and collagen I on days 7 and 14 after the UUO surgery (Figures 3A and 3B). Furthermore, rhALR was shown to significantly down-regulate the protein expression of vimentin and α-SMA in the obstructed kidneys by laser confocal immunofluorescence-staining analysis (Figure 3C). Then we explored the effects of rhALR on the gene expression of vimentin, α-SMA, fibronectin and collagen I in the sham-operated and obstructed kidneys 14 days after UUO surgery using RT–PCR. Figure 4 showed that compared with those in sham control, the mRNA expression of vimentin, α-SMA, fibronectin and collagen I were up-regulated in UUO rats treated with PBS, whereas rhALR administration significantly reduced the elevation of mRNA expression of these genes.

**Figure 5:**
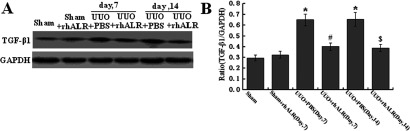
Effects of rhALR on the expression of TGF-β1 in UUO rats (**A**) The administration of 200μg/kg/day rhALR alone had no effect on the expression of TGF-β1 in sham-operated kidney, but significantly suppressed the up-regulated expression of TGF-β1 induced by UUO operation in obstructed kidneys on days 7 and 14 after surgery. (**B**). Graphical presentation of relative expression of TGF-β1. Values are presented as the density of TGF-β1 versus GAPDH (%). Mean±S.D. (*n*=6) were obtained from densitometric analysis of all individual experiments. *P<0.05 versus Sham group. #*P*<0.05 versus UUO+PBS (day 7). $*P*<0.05 versus UUO+PBS (day 14).

### Effects of rhALR on TGF-β/Smad signalling in UUO rats

**Figure 6:**
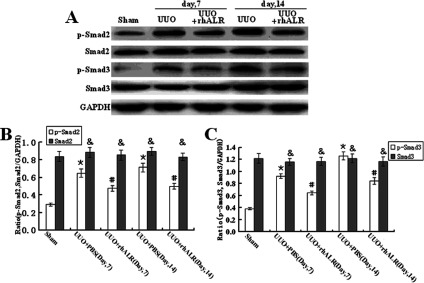
Effects of rhALR on the phosphorylation of Smad2 and Smad3 in UUO rats (**A**). The administration of 200μg/kg/day rhALR significantly suppressed the phosphorylation of Smad2 and Smad3 in obstructed kidneys in rats on days 7 and 14 after UUO. (**B**) Graphical presentation of relative expression of Smad2 and phosphorylated Smad2. (**C**) Graphical presentation of relative expression of Smad3 and phosphorylated Smad3. Values are presented as the density of target proteins versus GAPDH (%). Mean±S.D. (*n*=6) were obtained from the densitometric analysis of all individual experiments. &P>0.05 versus Sham group.*P<0.05 versus Sham group. #*P*<0.05 versus UUO+PBS.

To elucidate the potential molecular mechanism underlying rhALR's anti-fibrotic role in the rat UUO model, we further investigated the conventional TGF-β1/Smad signalling pathway in the progression of renal fibrosis. The UUO operation significantly up-regulated the expression of TGF-β1 in the obstructed kidney, and the administration of rhALR alone had no effect on the expression of TGF-β1 in the sham-operated kidney; however, rhALR dramatically suppressed UUO injury-induced TGF-β1 expression. Furthermore, the UUO operation had no effect on the Smad2 and Smad3′s protein expression in the kidney, while it significantly activated the phosphorylation of Smad2 and Smad3 on days 7 and 14 after the surgery. Moreover, the administration of 200 μg/kg/day rhALR could significantly suppress the phosphorylation of Smad2 and Smad3 in the obstructed kidneys in rats underwent UUO surgery, as shown in the Western blot analysis (Figure 6).

## DISCUSSION

The expression of ALR changes as the pathological pattern changes during the course of renal diseases. We previously evaluated ALR's expression in ischaemic and drug-induced AKI (acute kidney injury), and had proven ALR protected renal function by enhancing renal tubular epithelial cell regeneration [19–25]. ALR also exerted the anti-apoptotic effect on renal tubular epithelial cell in renal ischaemia reperfusion injury [20].

In this study, we investigated the effects of rhALR on the renal tubulointerstitial fibrosis induced by UUO in rats. Our observation also demonstrated that endogenous ALR was marginally detected in the tubulointerstitial and almost undetected in the glomerulus in the sham-operated kidney. ALR was involved in the pathological progression of renal fibrosis in the obstructed kidney in UUO rats. The expression of endogenous ALR increased gradually during the early phase of kidney obstruction (<7 days), and then decreased gradually to return to the original state on day 14 after the UUO surgery. In UUO rats model, the longer the obstruction existed, the more renal pathological features such as increased cell proliferation and ECM occurred in renal fibrosis. In the literature reported previously, the expression of vimentin and α-SMA was considered specific markers for fibroblasts and myofibroblasts that may have been derived from EMT (epithelial–mesenchymal transition). The fibroblasts and myofibroblasts are found in the interstitium of obstructed kidneys and such cells might produce ECM components, for example, collagen I, collagen III and fibronectin, similar to the components observed in the rat UUO model [26]. Our study showed that upon the administration of rhALR, the expression of proteins related to renal fibrosis, such as vimentin, α-SMA, collagen I and fibronectin, were reduced in obstructed kidney, whereas epithelial marker proteins, such as E-cadherin, was increased in obstructed kidney. Furthermore, rhALR inhibited vimentin, α-SMA, fibronectin and collagen I at transcription level. Our data also indicated that rhALR ameliorated renal fibrosis induced by UUO in a dose-dependent manner, and that rhALR played a role of anti-fibrosis in obstructed kidney. We conjectured that the increased expression of endogenous ALR represented a protective action induced by UUO, this protective action would gradually recede as the injury developed and ultimately led to renal fibrosis *in vivo*. Renal fibrosis is a shared pathological process of a wide variety of renal diseases, which eventually lead to end-stage renal disease [3]. TGF-β1 has been regarded as a key mediator in the progression of renal fibrosis [27]. TGF-β1 is up-regulated in the UUO model, which is associated with tubular EMT and increased synthesis of ECM [28]. Increasing evidence shows that TGF-β1 induces renal fibrogenesis by activating interstitial fibroblasts, myofibroblasts and tubule epithelial cells [26,29], and increasing ECM proteins [30]. TGF-β1 also mediates renal fibrosis by inducing the transformation of TECs (tubular epithelial cells) to myofibroblasts through EMT [31]. Other evidence has indicated that the fibrotic effect of TGF-β1 is mediated by a heteromeric receptor complex of the type I and II receptors, which activates the downstream intracellular mediators Smad2 and Smad3 via phosphorylation [28,32]. The pSmad-2 and pSmad-3 associate to form a heteromultimer with Smad-4 (Co-Smad). This complex is then translocated to the nucleus, where it can regulate the target gene expression [33,34]. Furthermore, TGF-β signalling has been shown to play a critical role as a potent fibrogenic inducer in renal fibrosis [29,35]. Therefore the targeted inhibition of signalling pathways involved in renal fibrosis is a promising therapeutic strategy for the treatment of fibrotic kidney diseases [36–39].

In this study, our observation indicated that TGF-β1 is up-regulated in the UUO model, as reported previously [28], and revealed that rhALR significantly suppressed the up-regulated expression of TGF-β1 induced by UUO operation in the obstructed kidney. We had demonstrated that the obstructed injury induced by UUO activated the phosphorylation of Smad2 and Smad3 protein and subsequently activated the downstream signalling of TGF-β/Smads pathway in UUO rats. The exogenous ALR (rhALR) inhibited Smad2 and Smad3 phosphorylation in UUO rats, which served a possible molecular mechanism underlying the antifibrotic capacity of ALR in renal fibrosis.

In conclusion, we studied the effects of the UUO-induced injury on endogenous ALR expression *in vivo* and showed ALR's involvement in the renal disease progression. Moreover, we also demonstrated that exogenous rhALR significantly ameliorated renal fibrosis in the obstructed kidney by inhibiting TGF-β/Smad activity. These findings may be contributed to further investigate ALR as an effective antifibrotic strategy to reverse renal interstitial fibrosis for CKD.
